# Draft Genome Sequence of *Lactobacillus farciminis* NBRC 111452, Isolated from Kôso, a Japanese Sugar-Vegetable Fermented Beverage

**DOI:** 10.1128/genomeA.01514-15

**Published:** 2016-01-14

**Authors:** Tai-Ying Chiou, Kenshiro Oshima, Wataru Suda, Masahira Hattori, Tomoya Takahashi

**Affiliations:** a ARSOA Research & Development Center, AOB Keioh Group Corporation, Hokuto, Yamanashi, Japan; b Department of Computational Biology, Graduate School of Frontier Sciences, The University of Tokyo, Kashiwa, Chiba, Japan; c Department of Microbiology and Immunology, Keio University School of Medicine, Shinjuku, Tokyo, Japan; d Cooperative Major in Advanced Health Science, Graduate School of Advanced Science and Engineering, Waseda University, Shinjuku, Tokyo, Japan

## Abstract

Here, we report the draft genome sequence of the *Lactobacillus farciminis* strain NBRC 111452, isolated from kôso, a Japanese sugar-vegetable fermented beverage. This genome information is of potential use in studies of *Lactobacillus farciminis* as a probiotic.

## GENOME ANNOUNCEMENT

Kôso is a popular sugar-vegetable beverage in Japan, with ingredients that include over twenty kinds of vegetables, fruits, and sugars. Wild lactic acid bacteria and yeasts cause it to ferment spontaneously (1, 2). Kôso is regarded as a health food, due in part to its functionalities that include anti-oxidative activity and a protective effect on ethanol-induced damage to gastric mucosa (2). The bacterial community in kôso changes and develops as fermentation proceeds. *Lactobacillus farciminis* NBRC 111452 was isolated from 45-day fermentate. As a nitric oxide donator, this species appears to be promising for use in probiotic treatment of gastrointestinal pathologies (3–6). It is also the first lactobacillus reported to have a rare type of C69-family dipeptidase, which has hydrolytic activity against Gly-Pro, a hard-to-degrade peptide that is produced during the enzymatic degradation of collagens (7).

Whole-genome sequencing of *L. farciminis* NBRC 111452 was performed using an Ion Torrent PGM system. A total read of 1,326,655 bp was assembled using Newbler v2.8 (Roche) into 58 contigs with *N*_50_ lengths of 70,772 bp. The resulting draft genome sequence is 2,354,209 bp with 125.7× redundancy and an average G+C content of 35.65%. The draft genome of *L. farciminis* NBRC 111452, annotated by the RAST server using Glimmer3, contains 2,491 candidate open reading frames, three rRNA genes, and 51 tRNA genes.

We identified protein-coding genes related to the reduction of nitrogen-containing compounds such as nitrite reductase (EC 1.7.2.2) and nitric oxide reductase (EC 1.7.2.5). The coding sequences of the arginine deiminase (EC 3.5.3.6) gene were also annotated. The gene for prolidase (EC 3.4.13.9), which plays an important role in the resynthesis of collagen, is also present in the genome. Nitric oxide has been reported to stimulate the activity of prolidase by increasing serine/threonine phosphorylation (8, 9), and the gene for serine/threonine protein kinase (EC 2.7.11.1) was also annotated in the genome. Annotation of the genome suggested that *L. farciminis* has multiple abilities to metabolize nitrogen-containing products. This information will assist probiotic studies.

### Nucleotide sequence accession numbers.

The genome sequence of *L. farciminis* NBRC 111452 has been deposited in DDBJ/EMBL/GenBank under accession numbers BCLJ01000001 to BCLJ01000058.
